# Chemo assist for children mobile health application to manage chemotherapy-related symptoms in acute leukemia in Indonesia: a user-centered design approach

**DOI:** 10.1186/s12887-023-04076-0

**Published:** 2023-05-30

**Authors:** Dwi Novrianda, Elisabeth Siti Herini, Fitri Haryanti, Eddy Supriyadi, Lutfan Lazuardi

**Affiliations:** 1 Department of Pediatrics and Maternity Nursing, Faculty of Nursing, Universitas Andalas, Padang, Indonesia; 2 Department of Child Health, Faculty of Medicine, Public Health and Nursing, Universitas Gadjah Mada/Dr. Sardjito Hospital, Yogyakarta, Indonesia; 3 Department of Pediatric-Maternity Nursing, Faculty of Medicine, Public Health, and Nursing, Universitas Gadjah Mada, Yogyakarta, Indonesia; 4 Department of Health Policy and Management, Faculty of Medicine, Public Health, and Nursing, Universitas Gadjah Mada, Yogyakarta, Indonesia

**Keywords:** Chemotherapy-related symptoms, Mobile health, Literature review, User-centered design, Symptom management

## Abstract

**Background:**

A mobile health (mHealth) application can encourage parents and pediatric patients to be involved in caring for their child’s health condition by providing the ability to identify and actively manage chemotherapy-related symptoms in their child. Several monitoring systems available today are diverse in features and system basis. This study aimed to develop and trial the Chemo Assist for Children (CAC) mHealth application for symptom management in children with acute lymphoblastic leukemia (ALL).

**Methods:**

In this study, the development of the CAC application went through multiple phases and methods. Study phases included: (1) development of the application’s feature based on the need assessment, (2) creation of content of application based on literature review, (3) develop prototyping of CAC, (4) expert review and feedback on the application content, (5) usability testing by targeted end-user.

**Results:**

Based on need assessment, it was determined that parents with leukemia children were interested in symptom management of chemotherapy and preferred mobile applications. Therefore, a mHealth application was designed to include features to identify symptoms and provide recommendation strategies to manage the symptom. Usability evaluation by end-user revealed that mHealth is a valid, accessible, and appropriate application for users.

**Conclusions:**

The CAC mHealth application developed can meet the needs of technology users to identify symptoms and manage chemotherapy-related symptoms in children with ALL. The CAC mHealth application can accommodate data not recorded at out-of-hospital care, increase the independence of symptom management, and improve communication between parents of children with ALL and health workers.

**Supplementary Information:**

The online version contains supplementary material available at 10.1186/s12887-023-04076-0.

## Introduction

Acute lymphoblastic leukemia (ALL) is a malignancy that attacks blood-forming tissues, most found in children, about 74% [1–3]. Global Cancer Statistics (2018) noted that the prevalence of leukemia in all countries was 2.4% of new cases and 3.2% of deaths. The increase in cancer treatment, namely chemotherapy, has shown an increase in survival rates, reaching more than 80% in developed countries [4, 5]. Indonesia is one of the top 30 countries with the most significant and rapid rise in age-standardized incidence rate (ASIR) in 2017 compared to 1990 (33,37%) [6].

Chemotherapy is considered effective in treating leukemia because it can maintain and contain the spread of cancer cells, slow the growth of cancer cells, kill cancer cells, and reduce symptoms caused by cancer [2]. However, chemotherapy-related symptoms in children with leukemia are individual and unique. Monitoring symptoms based on the child’s feelings and providing effective symptom management are essential aspects [7]. Moreover, it can also aid in overcoming chemotherapy-related symptoms [7, 8], shortening the treatment period, and preventing death [8], while improving children’s psychosocial well-being and quality of life [9].

Recently, the rapid development of technology in the health/nursing sector and the pandemic of COVID-19 have amplified the need for user-friendly mobile health (mHealth) applications, allowing for real-time monitoring for parents and children with ALL to benefit and facilitate them in long-term care and treatment. In pediatric cancer health services, several countries in the world have developed single or multiple symptom monitoring and management applications, including web-based [10–12], smartphones [13, 14], and computers [15]. Mobile health includes technologies such as mobile phones, personal digital assistance (PDA), smartphones, patient monitoring devices, mp3 players for mobile learning, and mobile computing [16]. A scoping review reports that health-based technologies need to be developed scientifically through theory, user needs, interests, and testing [17]. Therefore, we developed a mobile health-based symptom management intervention called Chemo Assist for Children (CAC) as the first step in monitoring and symptom management interventions to increase knowledge, self-management of symptoms, and quality of life in acute lymphoblastic leukemia children. The aims of this study are 1) to describe the process of developing a mobile health-based chemotherapy symptom management intervention program for children with acute lymphoblastic leukemia and 2) to evaluate CAC’s usefulness in improving the monitoring and symptom management in children with ALL.

## Methods

### Design

**Fig. 1:**
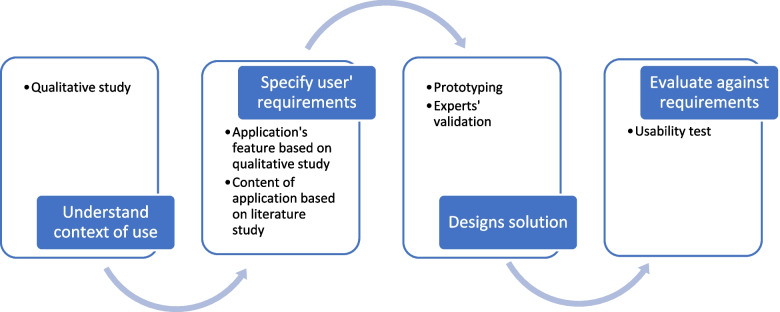
User-centred design of mHealth application CA C

This research was established in 4 stages (Fig. 1) and was guided by a user-centered design approach.

The type of research applied was research and development*,* which aimed to design and develop a mobile application to manage chemotherapy-related symptoms in children with ALL using an approach that involved a user-centered design and an extensive literature review.

#### Stage 1. Understand the context of the use

Through a qualitative study, phase 1 was carried out to understand the context and needs of the mHealth application users. Individual or group interviews using a semi-structured guide were conducted on 31 parents of children with acute lymphoblastic leukemia. Participants were recruited from the pediatric cancer treatment room at Dr. Sardjito Hospital Yogyakarta, and Dr. M. Djamil Hospital Padang used a purposive sampling method with the following inclusion criteria: parents who have children aged 0–18 years who are undergoing chemotherapy in the induction, consolidation, maintenance, and relapse phases.

Nine individual interviews and 2 group interviews with a maximum of 2 participants at Dr. M Djamil Hospital Padang and three group interviews at Dr. Sardjito Hospital Yogyakarta are performed and last about 1–1.5 h. All interviews use Indonesian. The first researcher and a research assistant facilitated the interview. The first researcher explained the purpose of the interview and asked participants’ opinions regarding the need for the mHealth application. The interview questions are provided in Additional file 1: Appendix 1.

The method for assessing user needs in the first stage is thematic analysis [18]. First, the coder read the transcript repeatedly and independently to identify meaningful statements from the participants. Second, they define statements describing the research objectives to be reduced to code. Third, codes that have similarities are collected into one category. Fourth, the interrelated categories give rise to a theme.

#### Stage 2. Specify user’s requirements

Software/application development requirements are collected and analyzed in this phase to produce complete requirements specifications. This activity is carried out as a basis for designing mHealth application products. The design of mHealth was developed based on literature studies. The researcher conducted a literature study to review chemotherapy-induced symptom questionnaires, quality of life, and symptom management due to chemotherapy based on scientific evidence published in textbooks and research journal articles. After that, the mHealth design is outlined in prototyping by web-based and mobile application programmer.

#### Stage 3. Designs solution

The content of the mHealth CAC application was reviewed by four experts, namely in child health and oncology, child health, pediatric oncology nursing, and psychology, and one expert in the IT field using a modified Delphi technique. The experts obtained a mock-up prototyping link, a content design module for the mHealth application, and a content validation assessment sheet. The researcher contacted the experts and provided information about the content validation procedure for the mHealth CAC application via email.

The researcher designed this assessment sheet and consulted with the supervisor (EHS, FH, ES). It consists of 15 statements including completeness of demographic data, 15 symptoms, the input of body temperature data and anthropometry (weight, height, mid-upper arm circumference), filling in daily child activity data, how to calculate caloric needs of children, management of nausea or vomiting, management of fluids and nutrition, management of mucositis, management of physical activity and rest, management of fever and neutropenia, management of relaxation, medication, monitoring charts, information on leukemia, and chemotherapy. The experts give an assessment in the form of a score of 1 for the appropriate statement or a score of 2 if it is not appropriate. Experts provide input and suggestions for any inappropriate statements in the column provided to improve the mHealth application content design.

If there is a difference of opinion among the experts, clarification efforts are made to reach an agreement through follow-up discussions via email and a short message service (SMS). Experts provide recommendations on the content of the mHealth application, including being suitable for use without revisions, ideal for use with modifications, and not suitable for use. Experts assess the mHealth application content design using the content validity assessment sheet twice. Experts provided input and scores on the mHealth application material in the first test. Then, the expert reviews the revision of the material and assesses its validity in the next test.

Data analysis in the expert validation phase descriptively explained the number of scores or the proportion of statements approved by the experts. Then a validity test was done by determining the content validity index (CVI) value. CVI is done in two steps, namely 1) calculating individual items (i-CVI) and 2) adding up the total expert scores (s-CVI). The CVI assessment for new instruments is recommended to have a minimum value of 0.8, declared valid. If the value is in the range of 0.7–0.79, it is recommended to be revised, and if it is less than 0.7, then the item is eliminated [19].

After the expert validates the application content, a consultation on the use of language in the application material is conducted with a linguist from the Indonesian Language Study Program, Faculty of Cultural Sciences, Universitas Gadjah Mada, to obtain adequate and efficient use of sentences in an application. After receiving approval for language validation, the next step was to prototype a mHealth application that could be accessed on mobile phones and subjected to usability testing.

#### Stage 4. Evaluate against requirements

Usability evaluation was conducted on ten parents of ALL children at two pediatric cancer referral hospitals in Indonesia. Parents of children with acute lymphoblastic leukemia undergoing chemotherapy in the induction, consolidation, and maintenance phases were recruited by convenience sampling. The usability evaluation used actions, behaviors, and commentary (ABCs) observations and completed a satisfaction questionnaire. The ABCs observation sheet method was used to observe 12 actions with four options of observation, namely 0 (very difficult) to 3 (no difficulty), 12 behaviors with 2 observation options, namely 0 (no presence) and 1 (presence), and comments shown by respondents while using the mHealth application. In this study, we used 4 points to observe parents’ actions using the mHealth application quickly. Likert scales have been used with different measurement ranges in terms of the number of answer choices from 2 to 11 points, where the shorter the scale (2 points, 3 points, 4 points), the higher the preference for the faster it is used. Meanwhile, the scale with more answer choices received a higher rating from the respondents concerning the adequacy of feelings expressed [20].

In this phase, the researcher met the respondents, explained the purpose of the usability evaluation, and obtained approval before participating. Respondents were asked to use this application for three days during hospitalisation. The IT team monitored the use of the mobile health application by respondents through the data server and reported it to the researcher (DN). In the observation method, one of the authors (DN) observed users directly on the first day with the help of an observation worksheet. The user is given an instruction sheet to run the application commands. Then the observer assessed the form in the domain of the action, and there was/no presence in the behavior’s domain.

Users are asked to fill out a satisfaction questionnaire at the end of using the mHealth application. This questionnaire was developed based on the content of the mHealth CAC application, which consists of 15 statement items. This questionnaire was reviewed by four experts (pediatric hemato-oncology, child health, nursing, and psychology) and one language expert from UGM. Respondents can choose from 1 to 5, where one means unsatisfactory, while 5 means very satisfactory. Users can add comments and suggestions for the mHealth application in this stage of development.

### Population and sample

At the application design development stage, we involved five experts in different fields, including a hemato-oncology pediatric clinician, a child health clinician, a psychologist, a pediatric nurse, and an information and technology specialist*.* Research subjects were selected based on their respective expertise to obtain content validity. Next, the trial phase involved ten parents of children with ALL who underwent chemotherapy at two teaching hospitals in Indonesia.

### Ethical considerations

This research has obtained ethical approval from the Medical and Health Research Ethics Committee of the Faculty of Medicine, Public Health, and Nursing, Universitas Gadjah Mada (KE/FK1007/EC/2020), and the Health Research Ethics Commission Dr. M. Djamil Hospital Padang (262/KEPK/2020). Permission and approval to conduct the study were received from the President Director of Dr. M. Djamil Hospital, Padang, and Dr. Sardjito Hospital, Yogyakarta, Head of the Installation of Pediatric and Midwifery Inpatient Dr. M. Djamil Hospital, Head of Installation of Estella Dr. Sardjito Hospital. Before the usability test, the researchers explained the aim of the study, the confidentiality of information, and the participant’s right to withdraw at any time. All participants in this study were parents of children with acute lymphoblastic leukemia, and no children under 16 years of age were involved. All parents or guardians obtained an informed consent form for audio-recording and using excerpts in publications and reports before the test. The participants were anonymous during the analysis and presentation of results.

## Results

### Stage 1. Understand the context of the use

The characteristics of the participants are summarized in Table 1. Participant feedback regarding the needs of the mHealth application includes 1). The need for effective and efficient communication media; 2) Requirements overview of the mHealth application. One parent expressed support for the existence of the mHealth app as follows:

*“I want to use an application because it’s simpler. Maybe there is an alternative, and there is a choice, right? There is an option if there is a book for those who are used to manuals. Then if there is an application, that means for those who are lazy to fill in data manually, and it might be complicated, it can be used online like Apps.” (Mother, 38 years old, Group Interview 2)*

**Table 1:** Characteristics of participants in stage 1 (*n* = 31)

No	Role	Sex	Age, years	Education	Chemotherapy’s phase
Group Interview 1, Dr. Sardjito Hospital Yogyakarta					
1	Mother	Female	35	Bachelor degree	Relapse induction
2	Mother	Female	32	Senior high-school	Relapse induction
3	Mother	Female	35	Senior high-school	Relapse induction
4	Mother	Female	30	Senior high-school	Relapse induction
5	Father	Male	40	Bachelor degree	Relapse induction
6	Father	Male	34	Senior high-school	Relapse induction
Group Interview 2, Dr. Sardjito Hospital Yogyakarta					
1	Mother	Female	36	Academy	Maintenance
2	Mother	Female	38	Bachelor degree	Maintenance
3	Father	Male	33	Bachelor degree	Maintenance
4	Father	Male	38	Senior high-school	Maintenance
5	Mother	Female	43	Senior high-school	Maintenance
6	Mother	Female	40	Junior high-school	Maintenance
Group Interview 3, Dr. Sardjito Hospital Yogyakarta					
1	Father	Male	34	Senior high-school	Induction
2	Father	Male	48	Junior high-school	Induction
3	Father	Male	40	Elementary school	Induction
4	Mother	Female	29	Junior high-school	Induction
5	Mother	Female	42	Senior high-school	Induction
6	Mother	Female	32	Bachelor degree	Induction
Individual Interview, Dr. M. Djamil Hospital Padang					
1	Mother	Female	45	Bachelor degree	Maintenance
2	Mother	Female	29	Bachelor degree	Maintenance
3	Father	Male	38	Bachelor degree	Consolidation
4	Mother	Female	31	Elementary school	Relapse induction
5	Mother	Female	31	Senior high-school	Relapse induction
6	Mother	Female	36	Junior high-school	Relapse induction
7	Mother	Female	43	Elementary school	Induction
8	Mother	Female	36	Academy	Induction
9	Mother	Female	38	Senior high-school	Induction
Group Interview 5, Dr. M. Djamil Hospital Padang					
1	Mother	Female	43	Senior high-school	Induction
2	Mother	Female	38	Junior high-school	Induction
Group Interview 6, Dr. M. Djamil Hospital Padang					
1	Mother	Female	42	Academy	Induction
2	Mother	Female	32	Bachelor degree	Induction

In addition, participants freely describe and convey their experiences with media that helps manage symptoms due to chemotherapy. Emerging themes, categories related to mHealth application needs, and other comments representing individuals or group interviews are presented in Additional file 2: Appendix 2.

### Stage 2. Specify user requirements

**Fig. 2:**
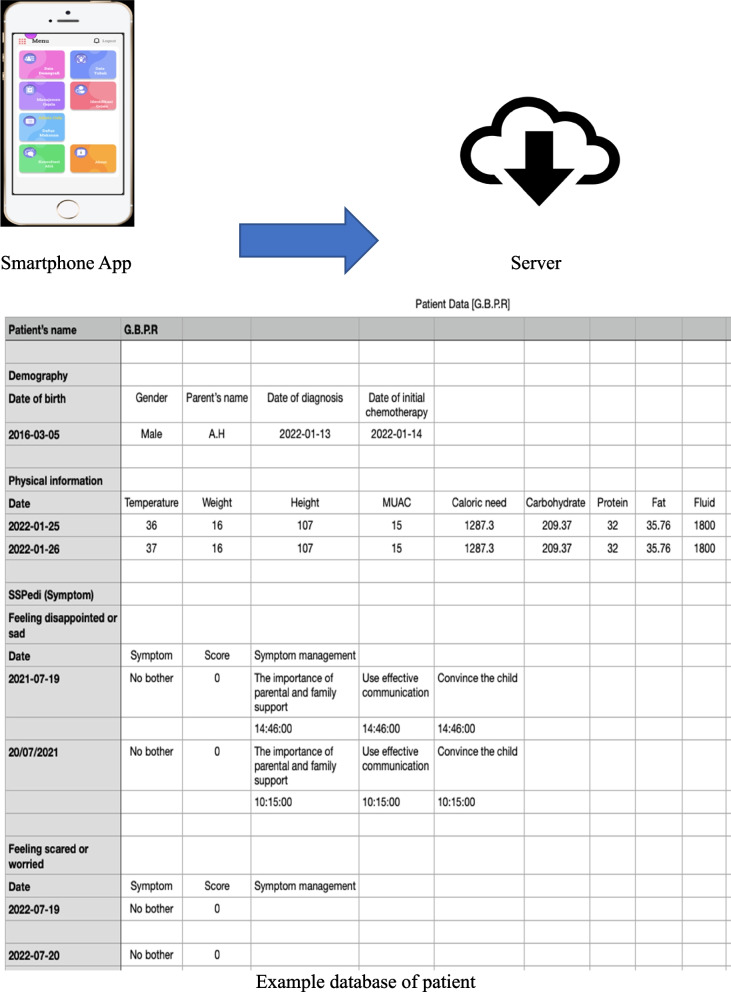
Database structure

**Fig. 3:**
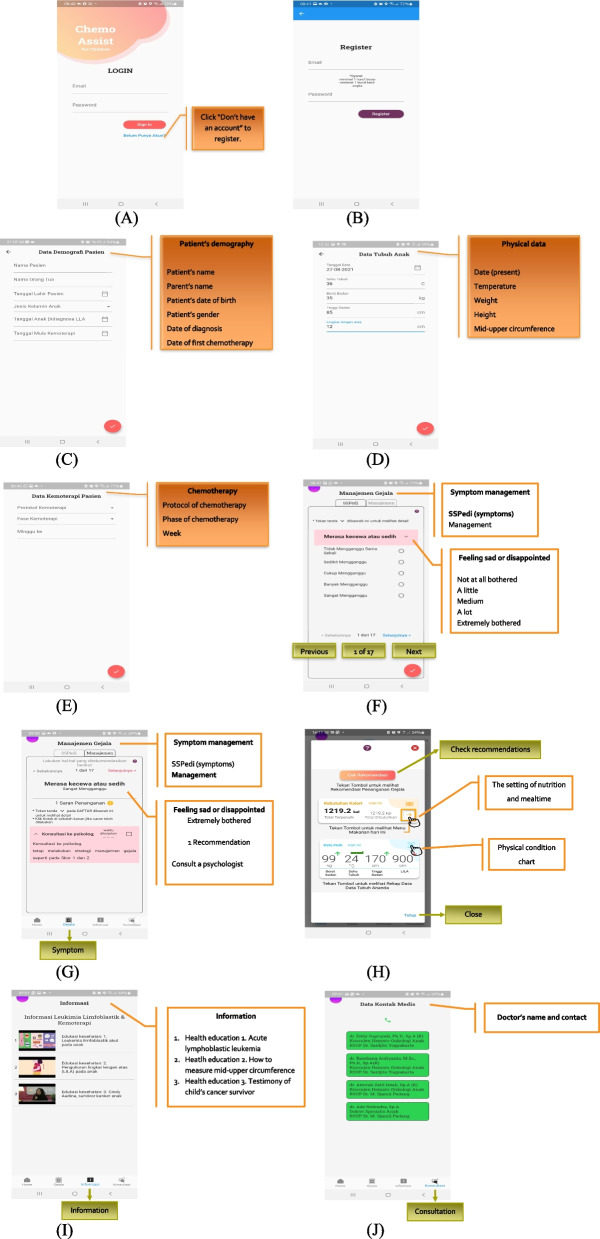
mHealth functionalities. **A**, **B** Log in page, **C** Patient’s demography, **D** Physical data, **E** Chemotherapy data, **F** Symptom management: Symptom identification using SSPedi, **G** Symptom management: recommendation of management, **H** Home page: check recommendation, nutrition, and mealtime’s setting, physical chart, **I** Information, **J** On-line consultation

Based on the results of qualitative research, a mHealth application design was made in the form of prototyping containing the main features, namely symptoms, symptom management, information, and consultation (Table 2). An overview of the database structure and functionalities of the symptom management application due to chemotherapy in children with ALL developed by researchers can be seen in Figs. 2 and 3 below.

**Table 2:** Description of the development of mHealth application features based on user needs

Theme	Category	Feature development	Function
The effects of chemotherapy are varied, individual, and comprehensive	1. Effects of chemotherapy on physical symptoms 2. Effects of chemotherapy on psychosocial 3. Perception of chemotherapy effect	Symptoms	To monitor symptoms due to chemotherapy using the SSPedi instrument
Management of chemotherapy-related effects requires adjustment, creativity, and critical thinking from parents	1. Management of chemotherapy effects 2. Information-seeking behavior 3. Support system 4. Adaptive Coping	a. Symptom management b. Information c. Online consultation	To provide recommendations and information on managing chemotherapy-induced symptoms; To connect communication between patients and health workers, namely doctors
Chemotherapy side effects are often challenging and complex, which can be difficult and upsetting for parents	1. Challenges during childcare 2. Challenges of health facilities and personnel 3. Financial and family challenges 4. Information challenge	a. Symptom management b. Information c. Online consultation	To provide recommendations and information on managing chemotherapy-induced symptoms; To connect communication between patients and health workers, namely doctors
Information and communication are essential, so applications based on user needs are very important	1. The need for effective and efficient communication media 2. App feature requirements 3. Constraints of digital or mobile approach	User-friendly application system, free-app, confidentiality and data security (registration and login system)	To provide comfort, convenience, and perceived benefits during the use of the mHealth application

Furthermore, several literatures were searched to strengthen the content of chemotherapy-induced symptom management materials used in the mHealth application. Literature publications in the form of descriptive or clinical trials, systematic reviews, guidelines, and textbooks are included in this literature review. Electronic databases for searches include PubMed and Google Scholar. Search keywords had procedures, child, pediatric, cancer, neoplasm, leukemia, symptom management, and symptoms (such as nausea, vomiting, fever, neutropenia, fatigue, mucositis, oral mucositis, pain, and cognitive impairment). The search results obtained 18 relevant references for material on the mHealth application, including five textbooks and 13 journal articles (Table 3). Searching the literature, as described in Table 3, facilitates researchers in designing the content of the mHealth application.

**Table 3:** Results of the literature search for mHealth app content

Content	Source	Type of Source
Upper arm circumference measurement	[21]	Textbook
Calorie requirement calculation	[21–23]	Textbook
Calculation of fluid requirement	[21]	Textbook
Fulfilment of nutrition during illness in cancer children	[21, 24]	Text / eBook
Management of nausea or vomiting	[25–29]	Journal article
Nutrition management	[30]	Journal article
Management of fever and neutropenia	[31]	Journal article
Fatigue management	[32]	Journal article
Mucositis management	[33]	Journal article
Pain management	[34, 35]	Journal article
Management of cognitive disorders	[36]	Journal article
Signs and symptoms of acute lymphoblastic leukemia	[37]	Journal article
Chemotherapy in children with acute lymphoblastic leukemia	[38]	Journal article

The mHealth application content includes strategies for chemotherapy-related symptoms, consisting of feeling disappointed or sad, feeling afraid or worried, feeling irritated or angry, having problems thinking or remembering things, changes in the body or facial appearance, feeling tired, feeling sick or having mouth sores, headache, aches or pains (other than headaches), tingling or numbness in the hands or feet, vomiting or feeling like throwing up, feeling very hungry or less hungry than usual, changes in taste, constipation, and diarrhea. For each symptom, information is provided in the form of a brief and precise description of the symptom and a score of the severity of the bothersome symptoms. Symptom management strategies include independent, evidence-based strategies helpful in reducing or managing symptoms (Additional file 3: Appendix 3).

### Stage 3. Designs solution

At this stage, the researchers recruited experts who worked at Sardjito Hospital, Universitas Andalas, and the Indonesian Pediatric Nurses Association by sending an email asking for willingness to review the content of the mHealth application. Table 4 revealed that generally, the experts involved in designing the content of this application are male (4, 80%) and have doctoral education (3, 60%). This shows that the experts at the content validation stage of the mHealth application have experience in their fields, so the reviews provided by these experts can be justified scientifically. In the initial assessment, the mHealth application content was not considered valid, and the experts offered several suggestions and input to increase the validity of the application content. Researchers improve the contents of the application material following the recommendations and feedback submitted by experts. The results of the first revision of the application content were consulted again and declared valid and suitable for use by experts in the second assessment. Table 5 shows that the experts’ increase in the total validity index occurred at the end from 0.75 to 0.97.

**Table 4:** Characteristics of experts on content validation

No	Gender	Education	Expertise	Time of assessment
P1	Male	Doctoral, Subspecialist	Pediatric Hematology-oncology (Consultant)	3
P2	Male	Master, Specialist	Child Health	2
P3	Female	Master	Pediatric Nursing	2
P4	Male	Doctoral	Psychology	2
P5	Male	Doctoral	Information Technology	2

**Table 5:** Content validity according to experts (*n* = 5)

No	Statement	First Assessment		Last Assessment	
		**f**	**I-CVI**	**f**	**I-CVI**
1	Input demographic data as needed	4	0.8	5	1
2	Data input 15 symptoms due to chemotherapy using the SSPedi tools	4	0.8	5	1
3	Input body temperature, weight, height, and UAC	5	1	5	1
4	Input data for children’s daily activities	3	0.6	5	1
5	How to calculate a child’s calorie needs	4	0.8	5	1
6	Management of nausea or vomiting	3	0.6	4	0.80
7	Nutrition and fluid management	4	0.8	5	1
8	Mucositis management	3	0.6	5	1
9	Management of physical activity and rest (sleep)	4	0.8	5	1
10	Management of fever and neutropenia	4	0.8	4	0.80
11	Management of relaxation activities, hypnosis, and positive thoughts	4	0.8	5	1
12	Medication according to doctor’s prescription	4	0.8	5	1
13	Daily, weekly, monthly chart depiction as needed in monitoring patient’s health status	3	0.6	5	1
14	Acute lymphoblastic leukemia information	4	0.8	5	1
15	Chemotherapy information	3	0.6	5	1
	Total mean score (S-CVI) (SD)	0.75 (0.358)		0.97 (0.058)	

*SSPedi* Symptom screening in pediatric, *UAC* Upper arm circumference, *I-CVI* Item-content validity index, *S-CVI* Scale-content validity index, *SD* Standard deviation

Table 6 outlines the feedback and changes made to improve the content of the mHealth app. The researcher corrected the contents of the application material and was consulted again until it was declared feasible by the experts. Experts provided substantial input on almost all menu items of the application’s content, including demographic data, the addition of infection and bleeding to chemotherapy-induced symptoms, the use of tools and methods for filling in children’s body data, management of chemotherapy-induced symptoms, and information on leukemia and chemotherapy. Two animated videos and video testimonials of child cancer survivors with a total duration of 12 min and 25 s can be accessed on the mHealth CAC application and Dwi Novrianda’s youtube.

**Table 6:** Recommendations for the content of the material in the application

Expert	Statement	Suggestions
1, 2	Data input 15 symptoms due to chemotherapy using the SSPedi tools	• Added signs of infection and bleeding in the Symptoms menu • Displays a description of each symptom operationally
1, 2	Input body temperature, weight, height, and upper arm circumference (UAC)	• Using standardized nutritional status parameters and appropriate measuring tools in measuring children’s nutritional status • Added upper arm circumference measurement step
2, 5	Input data for children’s daily activities	• There is a warning sign if the data is not filled in
1, 2, 3, 4	How to calculate a child’s calorie needs	• Using the REE formula according to FAO/WHO/UNU • Implementation of applicable calorie and fluid needs for families • Calorie and fluid requirement formulas are obtained from data input by the user
1, 2, 3	Management of nausea or vomiting	• Added management of dehydration due to vomiting or mucositis
2	Nutrition and fluid management	• Adding menu recommendations according to patient needs and ready-to-use products available on the market
1, 2, 3	Mucositis management	• Create a symptom algorithm that can be treated at home or must be immediately taken to the hospital
2	Management of physical activity and rest (sleep)	• Adding material on identifying danger signs that cause fatigue, such as anemia, hypoglycemia, or electrolyte disturbances due to dehydration so that management takes the form of immediately taking them to the hospital • Adding data on signs and symptoms that are easily recognizable by parents for decreased physiological status (not laboratory results)
1, 3	Management of fever and neutropenia	• Immediately take to the hospital if fever or neutropenia • Added anal fissure management
2	Management of relaxation activities, hypnosis, and positive thoughts	• Relaxation management is practiced in the form of video examples
2	Medication according to doctor’s prescription	• Add an explanation of the type of drug that should be taken, the timing and risk of drug interactions with other foods/drinks/drugs
1	Daily, weekly, monthly chart depiction as needed in monitoring patient’s health status	• What does the presentation of a graph?
2	Acute lymphoblastic leukemia information	• Added explanation of invasive procedures
2	Chemotherapy information	• It uses layman’s terminology and is easy to understand

*REE* Resting energy expenditure, *FAO/WHO/UNU* Food agriculture organization/world health organization/united nations university

### Stage 4. Evaluate against requirements

Of the 12 parents who met, ten people were willing to participate in the trial of the CAC application. Almost all respondents were female (90%) and aged 20–40 years (90%). Most respondents’ education level was intermediate (50%), and they generally worked as housewives (80%). Respondents had children with an average age of 8.75 (2–13 years) and were male (80%), undergoing chemotherapy in the induction phase (80%), and diagnosed with acute lymphoblastic leukemia less than three months (50%) (Table 7).

**Table 7:** Characteristics of usability test respondents (*n* = 10)

Variable	Frequency	Percentage
Gender		
Male	1	10
Female	9	90
Age (year)		
20 – 40	9	90
More than 40	1	10
Education level		
High education (Academy/Bachelor/Postgraduate)	3	30
Middle education (High school)	5	50
Primary education (Elementary/Junior school)	2	20
Profession		
Working (Civil servants)	2	20
Not working (Housewife)	8	80
Child’s age (year) (median, min–max)	8.75 (2–13)	
Child’s gender		
Male	8	80
Female	2	20
Phase of chemotherapy		
Induction	8	80
Consolidation	1	10
Maintenance	1	10
Long-time diagnosed with ALL		
< 3 months	5	50
3 – 6 months	1	10
> 6 months – 1 year	0	0
A year – 3 years	1	10
> 3 years	3	30

Almost all respondents could complete the tasks in the trial well, for about 20–30 min. However, one respondent experiences application problems due to cellular network interference. Table 8 shows the observations on the first day of 10 parents with ALL children. In the Actions section, the average is 2.93 (maximum three and minimum 2). Almost all respondents did not experience difficulties in using symbols or icons in the application and using the mHealth CAC application. There was only one respondent who experienced quite a bit of problem and five respondents who had a little difficulty in recognizing the detail icons on the symptom management menu.

**Table 8:**
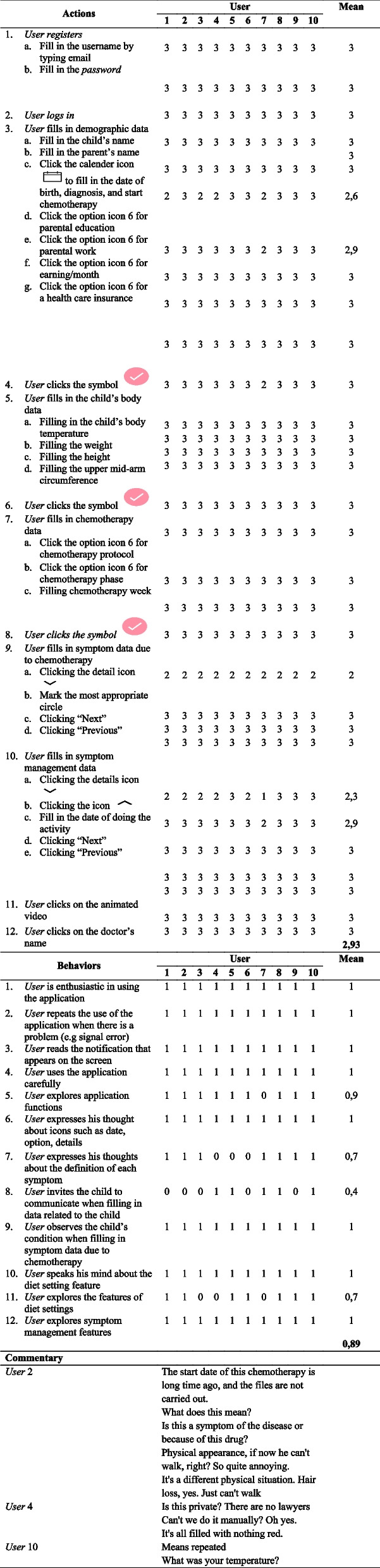
User observations using the ABCs method (*Actions, Behavior, and Commentary*)

Almost all respondents are interested in using and exploring the features contained in the application. However, only four people invited children to discuss filling out symptom data due to chemotherapy. Furthermore, in the Behaviors section, the average is 0.89 (maximum one and minimum 0.4).

Respondents’ assessment of the satisfaction of using the mHealth CAC application in the application trial after using the mHealth CAC application for three days can be seen in Table 9. The average total respondent satisfaction with the use of the CAC application is 4.28 (0.32), which means that respondents are satisfied with the CAC.

**Table 9:** Average respondents’ satisfaction with the use of the Chemo Assist for Children (*n* = 10)

Statement	Very dissatisfied	Dissatisfied	Enough satisfied	Satisfied	Very satisfied
1. Fill in the application according to the child’s needs	0	0	3	5	2
2. Fill in the application according to the doctor’s recommendation	0	0	0	8	2
3. Application presentation according to the times	0	0	0	4	6
4. Application helps in the identification of symptoms due to chemotherapy	0	0	1	6	3
5. Applications encourage the management of symptoms due to chemotherapy	0	0	0	6	4
6. The app’s learning method is easy to understand	0	0	1	5	4
7. The app’s symptom management is easy to understand	0	0	0	8	2
8. The language is easy to understand and clearly read	0	0	0	3	7
9. The terms in the app are understandable	0	0	3	4	3
10. Sentence descriptions or app commands are continuous and easy to follow	0	0	0	7	3
11. App features easy to understand	0	0	1	6	3
12. The size of the text reads well	0	0	0	5	5
13. Very interesting pictures	0	0	1	6	3
14. The activity flow is clear and interesting	0	0	1	5	4
15. Very attractive color	0	0	0	8	2
Total mean (SD)	4.28 (0.32)				

Most of the respondents gave a good impression of the CAC mHealth application. The application contains information that is easy to understand and provides benefits during the treatment of children. The following are comments from respondents:

“This application has increased our knowledge and helped us deal with our problems, especially in handling children’s health during the treatment/chemotherapy. Hopefully, this application will be easier for users to use in the future.” (R1)

“The app is good and easy to understand.” (R2)

“Quite understandable.” (R4)

“Good and helpful.” (R5)

“The application is easy to understand and easy to enter data.” (R10)

In addition to general comments, participants also gave specific suggestions for further improvement of the application, as follows:

“The application is easy to understand; there are only signal problems when using it.” (R1)

“The symptom question no. 1 focuses more on the patient’s condition; No. 5 added details in the form of physical conditions, not only physical appearance.” (R2)

“Pease activate for a doctor’s consultation while we are at home. For information, please increase the content that makes children enthusiastic about recovering, for example, children/adolescents who are drug-free/recovered.” (R10)

## Discussion

This study used the user-centered design to design, implement, and evaluate the CAC mobile health application. This application allows users to identify and manage symptom data due to chemotherapy in children with ALL in one place. Besides, it records children’s body temperature and anthropometric status to monitor possible fever or infection and decrease or improve nutritional quality. This application feature can encourage users to be more involved in their child’s health care by implementing chemotherapy-related symptom management recommendations and improving health care quality.

The CAC mHealth application was designed and developed to explore parents’ role in managing chemotherapy-related symptoms and the need for health application media for children with ALL and supplemented by a literature review of current studies. There are seven application modules: patient demographic data, primary health data, regulation of caloric and fluid requirements, symptom identification with *Symptom Screening in Pediatrics* (SSPedi) tools [39], chemotherapy-related symptoms, information, and consultation implemented into a mobile application. These modules make it easy for users to manage children’s health data with ALL. One study reported that two forms of worksheet-based intervention, namely symptom management and coping and support as parent education strategies for discharge in children with cancer, showed more significant pain reduction on symptom management worksheets and no difference in symptoms of nausea and appetite disturbances between the two forms of intervention. This study implies using electronic formats in symptom management [40].

As a result of reviews and feedback from experts, there were 20 feedbacks to improve the application content. Of the 15 symptoms on the SSPedi instrument, two other symptoms were added: infection and bleeding. Interestingly, this application is not only for one symptom but for multiple symptoms, namely 17 symptoms, so there are 17 symptom management spread into 41 items and 120 sub-items of recommended symptom management strategies due to chemotherapy. This differs from the C-TIPS and Pain Squad applications used to manage pain in children and adolescents with cancer. In addition, information features about ALL and chemotherapy, as well as testimonials on the healing of ALL survivors, which are available in a 12-minute animated video format, can provide insight into children’s disease and treatment and motivation to undergo chemotherapy treatment regularly. Using this mobile app to manage chemotherapy-induced symptom data flexibly can also fill the information gap between clinical visits. Mobile-based education effectively improves mothers’ knowledge and decisions in managing a child when choking [41]. Similarly, a critical review [42] found that using supportive technology for children and adolescents with chronic conditions increased their knowledge of the disease and its psychosocial aspects.

This CAC mHealth application is designed to be easy to use and user-friendly for parents and children. The individual pages in the CAC mobile application are designed to be simple and have a specific purpose, such as entering children’s chemotherapy data and identifying chemotherapy symptoms. Therefore, the end-users rated the usability highly in the instrument. Ten parents with diverse social, educational, and ethnic backgrounds tested the CAC mobile health application. This way, we could get various inputs and accommodate user needs with multiple characteristics. The CAC mHealth application users were involved throughout the application trial implementation process. During the procedure, users actively contribute by providing ideas and feedback on the prototyping version in usability tests. Suggestions and feedback from users at the pilot stage have been incorporated into the current version of the CAC mHealth application. Respondents felt generally satisfied and thought the application provided information, assistance, and solutions during chemotherapy treatment and treatment. Overall, the mHealth application was easy to understand and use.

With this CAC mobile app’s availability, users can easily manage all their health data in one place, including data during treatment and those generated between their typical clinical visits. These data are often not available to medical professionals. As a result, the information gap will be filled. Healthcare providers can obtain more reliable and comprehensive patient data, which can help them better understand the ineffectiveness of specific therapies. Healthcare providers can utilize information in their decision-making, increasing the quality of health services.

The application implementation design has also integrated several solutions for the barriers to adopting CAC. As obtained from the exploration findings, concerns about *cybercrime* and the possibility of internet signal/network constraints exist. Several mHealth security threats include malware infections, hackers, mobile phone theft, human users, and data theft by third parties such as insurance companies and intelligence departments [43]. The application has used strong security measures with password criteria of at least eight characters with a combination of upper- and lower-case letters. This feature will help the CAC mHealth application achieve higher adoption rates.

Meanwhile, to overcome internet network problems, this CAC mHealth application is designed to be used when an internet connection is unavailable or the network is in inadequate condition. Some users do not always have a strong network or internet signal, so any data that the users input is stored in advance on the mobile device or *Smartphone.* Then when users have a strong network, these data are automatically entered and uploaded into the application system. This action allows users not to need to repeat data entry. This finding aligns with the review [44] that internet connectivity is one of the barriers to using mHealth applications.

This research aimed not to create mobile applications to meet everyone’s needs but to build a mHealth application with a specialized purpose and provide the CAC mHealth application to specific users. This CAC mHealth app is designed to be extendable; therefore, it will be easier to add new features according to user needs and feedback. After users use it for a certain period, they will better understand ​​what further improvements may be needed.

### Limitations

Some limitations of our study deserve note. The generalizability of results may be limited by study sample, location, and inclusion/exclusion criteria. Our target population is parents with children with acute lymphoblastic leukemia undergoing chemotherapy in the induction and consolidation phases. The results may differ in other groups with children with ALL in the maintenance phase, other types of leukemia, and various locations. This research focuses on two provinces in Indonesia, West Sumatra, and Yogyakarta. However, our sample can represent parents of children with acute lymphoblastic leukemia in Indonesia. These two hospitals are regional referral hospitals for patients from neighboring provinces, such as North Sumatra, Jambi, Riau, West Java, Central Java, East Java, and the Moluccas. However, the possible unique aspects of chemotherapy-related symptom management at this site may limit the generalizability of the study’s findings.

The usability test instrument is a novel questionnaire made by researchers to assess respondents’ satisfaction with using the application from five aspects: material coverage and accuracy, recency, encouraging curiosity, clarity of material and language, and application attractiveness. In the observation method, the usability evaluation is carried out by one observer, so it is not possible to ignore the possibility of psychometric problems of observer effects and inter-observer reliability. Furthermore, the instrument used in evaluating usability is not an instrument that has been validated because the currently available questionnaire does not meet specific needs in evaluating CAC mHealth application content. This approach may miss some data in the collection, such as future application usage issues and obstacles encountered while using the application. The psychometric properties of the usability questionnaire have not yet been researched or validated. The questionnaire has not yet been applied to other products or systems or compared to other usability instruments. Therefore, the conclusions and generalizations that can be drawn from its use here are limited.

### Implications for further research

Future research needs to determine the efficacy and effectiveness of using the CAC mHealth application in clinical and community settings. Collecting qualitative data in evaluating user experience, benefits and challenges felt by users, and significant and less important features of the mHealth application is also vital to improve the application’s performance and quality.

### Implications for health services

The presence of the CAC mHealth application for children with ALL can fill in the gaps in data that were not evaluated while patients are out of the hospital. The availability of recommendations and information on symptom management due to chemotherapy in the CAC mobile application can further increase independence and empower parents and children. In addition, the application can accommodate communication between parents, children, and health practitioners in recognizing and dealing with symptoms that arise during chemotherapy.

## Conclusions

The CAC mHealth application was developed based on user needs and validated by experts to be functional and easy to use. Chemo Assist for Children is a mobile-based health application that supports parents of children with leukemia undergoing chemotherapy treatment. The support presented through the application is expected to overcome various problems that arise from physical, psychological, and social aspects.

## Supplementary Information

**Additional file 1: Appendix 1.** Interview guide.

**Additional file 2: Appendix 2.** Coding process, from meaning units to themes.

**Additional file 3: Appendix 3. **Symptom management strategies.

## Data Availability

The corresponding author’s data supporting this study’s findings are available upon reasonable request.

## Abbreviations

mHealth: Mobile health
CAC: Chemo assist for children
ALL: Acute lymphoblastic leukemia
SMS: Short message service
CVI: Content validity index
I-CVI: Item-Content validity index
S-CVI: Scale-Content validity index
App: Application
SSPedi: Symptom screening in pediatrics
UAC: Upper arm circumference
