# Supplementary material for: Chemo assist for children mobile health application to manage chemotherapy-related symptoms in acute leukemia in Indonesia: a user-centered design approach
Source: BMC Pediatr. 2023 May 30;23:274. doi: 10.1186/s12887-023-04076-0 (PMC10227782; doi:10.1186/s12887-023-04076-0)
Supplement: Supplementary file 1 — Additional file 1: Appendix 1. Interview guide. [file 12887_2023_4076_MOESM1_ESM.docx]

**Appendix 1.** Interview guide

1. What can help you obtain information in dealing with chemotherapy-related symptoms in your child?
2. What media (books, flipcharts/leaflets, Google, health applications) have been used to treat chemotherapy-related symptoms that your child is experiencing?
3. How was your experience using media to treat chemotherapy-related symptoms?
4. How suitable are media used to help you deal with chemotherapy-related symptoms?
5. What do you need regarding media use in dealing with chemotherapy-related symptoms in children?
6. What are your hopes for using media that has been used in dealing with chemotherapy-related symptoms in children?
7. What obstacles did you experience when using the media?
