# Supplementary material for: Chemo assist for children mobile health application to manage chemotherapy-related symptoms in acute leukemia in Indonesia: a user-centered design approach
Source: BMC Pediatr. 2023 May 30;23:274. doi: 10.1186/s12887-023-04076-0 (PMC10227782; doi:10.1186/s12887-023-04076-0)
Supplement: Supplementary file 2 — Additional file 2: Appendix 2. Coding process, from meaning units to themes. [file 12887_2023_4076_MOESM2_ESM.docx]

**Appendix 2** Coding process, from meaning units to themes

| **Meaning units** | **Codes** | **Categories** | **Themes** |
| --- | --- | --- | --- |
| "…Now I'm a bit embarrassed. This is a bad example. So, I missed a lot. I don't take it more often. I was told to take it back and forth, but I always didn't bring it. For the first time, it was often... hospitalized, yes, carrying it was heavy, a bit thick, and then I left it alone; after that, I always forgot. So I didn't put that in every lab result in that book; that's it. Maybe others are better at filling it in more diligently." (Mother, P2, Group 2 Interview)  "... Halodoc, we never used the application because we didn't believe he knew about our illness, but e the usage has been very high since Covid, and people can't leave the house right, e.. since the lockdown, Halodoc has become the most popular application. Ha, that's all the input." (Mother, P2, Group 2 Interview)  "Yeah, I think that's helpful. He 'em... to supervise e... I mean how do we need to control... our child's development…" (Fathers, P4, Group 2 Interview)  "If I do, yes, because it's simpler. Maybe e ... there are alternatives; there are options, right? There is an option, and if you are used to having a manual, you already have a book. If there is already one... already there, yes. application means… the models… those who are too lazy to do manual data… and it might be a hassle, you can use online ones like Apps… that's for me, ma'am." (Mother, P2, Group 2 Interview) | The need for patience in the use of pocketbooks  The Mobile Approach Depending on Pandemic Conditions  Digitization or virtual approach  Benefits of online or digital methods | The need for more effective and efficient communication media | Information and communication are important, so application-on-demand delivery apps are essential |
| "…Then, if you want, there is a possibility that if we usually have an application, we ask customer service, right? or ask something like that, maybe a notification can come out there, connect to an email or chat; if you chat it's better, you can answer it right away, or later you can call by phone to this number. So if someone is on guard, can provide information about the questions and maybe what questions can be given. He has standard questions; there are answers." (Mother, P2, Group 2 Interview)  "There are general tips for thousands because it turns out that the protocol in that group is still a lot of people who ask. So, when we enter when we want our children to be fed, we are given health protocols, too; that's how it is. …” (Mother, P2, Group 2 Interview)  "…But for example, if he wants to consult, maybe he can connect to the chat which dialed, so not all of them go directly to the experts, you know. It's the sort of questions, that also makes it easier. Then later... there was a doctor on duty today, so maybe if you want to ask about things that children sometimes do... parents who have children like this are a little worried. Yes, what cough... runny nose, that's just worrying. Coughing up a little sometimes... my child coughs, but rarely coughs. He only coughed a few times. Maybe with that... easy access, easy talking, which... doesn't... like bothering the doctor, right? (Mother, P2, Group 2 Interview)  "Hmm, for media applications, there's no problem. Hmm, I mean, I mean user-friendly, I made it as easy as possible…" (Mother, IDI 1)  "There's no payment, right? It's like an application; what's the name? It's a burden for us; we are already like this. Yes, what are the names of the tutors who pay for it, what is the teacher… Ruang Guru… Haa, you don't have to be like that… aa… like that aaa (Mother, P1, Group 5 Interview) | Customer care  Childcare protocols or general information  Online consultation  User-friendly  Free app | Application feature requirement |  |
| "Yes, it's easy, it's just that the problem is sometimes the signal" (Mother, P1, Group 4 Interview)  "…this is this application, isn't it? So I'm back again thinking about this cybercrime, isn't it? So we didn't meet the person, but we can reply to our conversation, spam, for example, right, so I'm not underestimating technology; technology is good, but in our society, do you often misuse the progress of technological science [oo I see] this technology was created to make it easier for humans, right [yes it's true]. Still, some people abuse it…." (Father, IDI 3). | Signal difficulty  Cybercrime concerns | Constraints of digital or mobile approach |  |
