# Supplementary material for: Chemo assist for children mobile health application to manage chemotherapy-related symptoms in acute leukemia in Indonesia: a user-centered design approach
Source: BMC Pediatr. 2023 May 30;23:274. doi: 10.1186/s12887-023-04076-0 (PMC10227782; doi:10.1186/s12887-023-04076-0)
Supplement: Supplementary file 3 — Additional file 3: Appendix 3. Symptom management strategies. [file 12887_2023_4076_MOESM3_ESM.docx]

**Appendix 3** Symptom management strategies

| **Symptoms** | **Symptom Management** | | |
| --- | --- | --- | --- |
|  | **Score 0 (Not at all bothered)** | **Score 1 (A little) dan 2 (Medium)** | **Score 3 (A lot) dan 4 (Extremely bothered)** |
| Feeling disappointed or sad | The importance of parental or family support (3 items) | Efforts to channel children’s feelings (7 items) | Immediately report to the health worker or take him to the hospital |
| Feeling scared or worried | The importance of parental or family support (3 items) | Efforts to channel children’s feelings (7 items) | Immediately report to the health worker or take him to the hospital |
| Feeling cranky or angry | The importance of parental or family support (3 items) | Efforts to channel children’s feelings (6 items) | Immediately report to the health worker or take him to the hospital |
| Problems with thinking or remembering things | The importance of parental or family support (3 items) | Activities to improve brain function (3 items) | Immediately report to the health worker or take him to the hospital |
| Changes in how your body or face look | Providing support in self-care (3 items) | Efforts to improve children’s appearance (5 items) | Immediately report to the health worker or take him to the hospital |
| Feeling tired | - | Efforts to overcome child fatigue (9 items) | Immediately report to the health worker or take him to the hospital |
| Mouth sores | Oral hygiene (4 items) | Oral care activities (5 items);  Nutritional support for children (6 items);  Report immediately with the indication (1 item) | Immediately report to the health worker or take him to the hospital |
| Headache | - | Report immediately with the indication (1 item);  Efforts to overcome pain/sick (3 items);  Medication according to doctor’s prescription (1 item) | Immediately report to the health worker or take him to the hospital |
| Hurt or pain (other than a headache) | - | Report immediately with the indication (1 item);  Efforts to overcome pain/sick (3 items);  Medication according to doctor’s prescription (1 item) | Immediately report to the health worker or take him to the hospital |
| Tingly or numb hands or feet | Medication according to doctor’s prescription (1 item) | Report immediately with the indication (1 item);  Motor and sensory exercises (5 items) | Immediately report to the health worker or take him to the hospital |
| Throwing up or feeling like you may throw up | - | Efforts to overcome nausea/vomiting (4 items);  Treatment of dehydration (2 items) | Immediately report to the health worker or take him to the hospital |
| Feeling more or less hungry than you usually do | - | Nutritional support for children (9 items);  Physical activity (1 item) | Immediately report to the health worker or take him to the hospital |
| Changes in taste | - | Nutritional support for children (7 items);  Treatment of dehydration (1 item) | Immediately report to the health worker or take him to the hospital |
| Constipation (hard to poop) | - | Nutritional support for children (5 items);  Anal care (1 item);  Medication according to doctor’s prescription (2 items) | Immediately report to the health worker or take him to the hospital |
| Diarrhea (watery, runny poop) | - | Report immediately with the indication (1 item);  Fluid and nutritional support (8 items);  Anal care (1 item);  Treatment of dehydration (1 item) | Immediately report to the health worker or take him to the hospital |
| Infection | Improvement of personal and environmental hygiene (2 items);  Visit restrictions (1 item);  Dental and oral hygiene (1 item);  Genital hygiene (1 item);  Use of masks (1 item);  Food hygiene (1 item) | Report immediately with the indication (1 item);  Medication according to doctor’s prescription (1 item) | Immediately report to the health worker or take him to the hospital |
| Bleeding | Nutritional support for children (1 item);  Recommended physical activity (1 item);  Use of a soft toothbrush (1 item);  Avoid certain drugs (1 item) | Report immediately with the indication (1 item);  Recommended physical activity (1 item);  Efforts to overcome nose bleeding (1 item);  Use of a soft toothbrush (1 item);  Medication according to doctor’s (1 item) | Immediately report to the health worker or take him to the hospital |
